# Apoptosis or Antiapoptosis? Interrupted Regulated Cell Death of Host Cells by Ascovirus Infection *In Vitro*

**DOI:** 10.1128/mbio.03119-22

**Published:** 2023-02-06

**Authors:** Huan Yu, Jin-Rong Li, Hua-Yan Xiao, Sheng-Kai Cao, Bin Chen, Ni Li, Gong Chen, Guo-Hua Huang

**Affiliations:** a Hunan Provincial Key Laboratory for Biology and Control of Plant Diseases and Insect Pests, Hunan Agricultural University, Changsha, Hunan, China; b College of Plant Protection, Hunan Agricultural University, Changsha, Hunan, China; Boston Children’s Hospital; Catholic University of America

**Keywords:** regulated cell death, ascovirus, apoptosis, pyroptosis, *Heliothis virescens* ascovirus 3h

## Abstract

Ascoviruses are insect-specific viruses thought to utilize the cellular apoptotic processes of host larvae to produce numerous virion-containing vesicles. In this study, we first determined the biochemical characteristics of ascovirus-infected, *in vitro-*cultured insect cells and the possible antiapoptotic capacity of ascovirus-infected insect cells. The results indicated that the ascovirus infection in the first 24 h was different from the infection from 48 h to the later infection stages. In the early infection stage, the Spodoptera exigua host cells had high membrane permeability and cleaved gasdermin D (GSDMD) but uncleaved Casp-6 (SeCasp-6). In contrast, the later infection stage had no such increased membrane permeability and had cleaved SeCasp-6. Four different chemicals were used to induce apoptosis at different stages of ascovirus infection: hydrogen peroxide (H_2_O_2_) and actinomycin D (ActD) had similar effects on the ascovirus-infected cells, whereas cMYC inhibitors and tumor necrosis factor alpha (TNF-α) plus SM-164 apoptosis inducers (T/S) had similar effects on infected cells. The former two inducers inhibited viral DNA replication in most situations, while the latter two inducers inhibited viral DNA replication in the early stage of infection but promoted viral DNA replication in the later infection stage. Furthermore, immunoblotting assays verified that T/S treatment could increase the expression levels of viral major capsid protein (MCP) and the host inhibitor of apoptosis protein (SeIAP). Coimmunoprecipitation assays revealed interaction between SeIAP and SeCasps, but this interaction was disturbed in ascovirus-infected cells. This study details the *in vitro* infection process of ascovirus, indicating the utilization of pyroptosis for antiapoptosis cytopathology.

## INTRODUCTION

Regulated cell death (RCD) is a set of genetically encoded mechanisms for the targeted elimination of superfluous, irreversibly damaged, and/or potentially harmful cells (1, 2). According to the Nomenclature Committee on Cell Death (NCCD), cell death can be classified according to the morphological appearance of the lethal process and includes apoptosis, necrosis, autophagy, and pyroptosis (3). Apoptosis is synonymous with “programmed cell death” (PCD), which is an expression that insinuates cell death has been genetically programmed and is not accidental (4).

The infection process of a virus and the defense process of its eukaryotic host cells are orderly and delicate processes (5). It is generally believed that eukaryotic cells can use their own apoptosis as an effective means to prevent infection by a virus or pathogenic microorganism (6, 7). To gain sufficient time and space for replication or assembly, viruses have evolved various ways to inhibit the apoptosis of host cells, among which the most common way is to encode highly efficient blockers, such as inhibitors of apoptosis proteins (IAPs) (8–11). For example, a field-collected Autographa californica nucleopolyhedrovirus mutant (AcMNPV-vAcAnh) was found to have deficient infectivity both *in vivo* and *in vitro* (12). A comparison of the genomic DNA sequences of the infection-deficient AcMNPV isolate and the infectious AcMNPV isolate revealed that only one gene was mutated in the latter (13). This deleted gene (*p35*), in baculovirus, was the first *iap* gene defined in insect viruses. The *p35* deletion mutant baculovirus can cause apoptosis of host cells immediately after infection, thus almost losing the ability to infect (11, 13–15).

Ascoviruses are large, circular, double-stranded-DNA insect viruses. Their pathogenesis has been reported as an apoptosis-like process, which can directly promote host cell apoptosis by using its coded caspase-like protein (16, 17). Therefore, ascoviruses use apoptotic bodies generated by this process to form vesicles and complete their own packaging. This unusual mode of pathogenesis is how the name “ascovirus” originated and also makes ascoviruses unique among DNA insect viruses (18). However, there are still many unknown aspects about the pathogenesis of ascoviruses, such as how they accurately control the activity of their coded caspase-like protein. It is also unknown whether the premature expression of this caspase-like protein, an early-expressed protein, results in a failed infection, as in the case of the *p35* deletion mutant baculovirus. In our previous study, transmission electron microscopy (TEM) was used to observe the RCD morphology of Heliothis virescens ascovirus 3h (HvAV-3h)-infected tissues of Spodoptera exigua larvae and Helicoverpa armigera larvae. No apoptotic criteria were found in any of the TEM images, but some necrosis and/or pyroptosis-like hemocytes were found in the early stage of infection (19). These results indicated that the *in vivo* pathogenesis of ascovirus infection was accompanied by complex host cellular death processes.

To investigate the relationship between ascovirus infection and host RCD, a series of validations at the level of *in vitro*-cultured cells were performed in this study. Given that HvAV-3h-infected cells are resistant to hydrogen peroxide-induced apoptosis, we further studied the relationship between ascovirus and host cell RCD at different infection stages. The results obtained in this study reveal the utilization of different types of RCD in host cells during ascovirus infection, which lays the foundation for further studies on the pathogenicity of ascoviruses.

## RESULTS

### Different RCD biochemical characteristics were found in different HvAV-3h infection stages *in vitro*.

**FIG 1:**
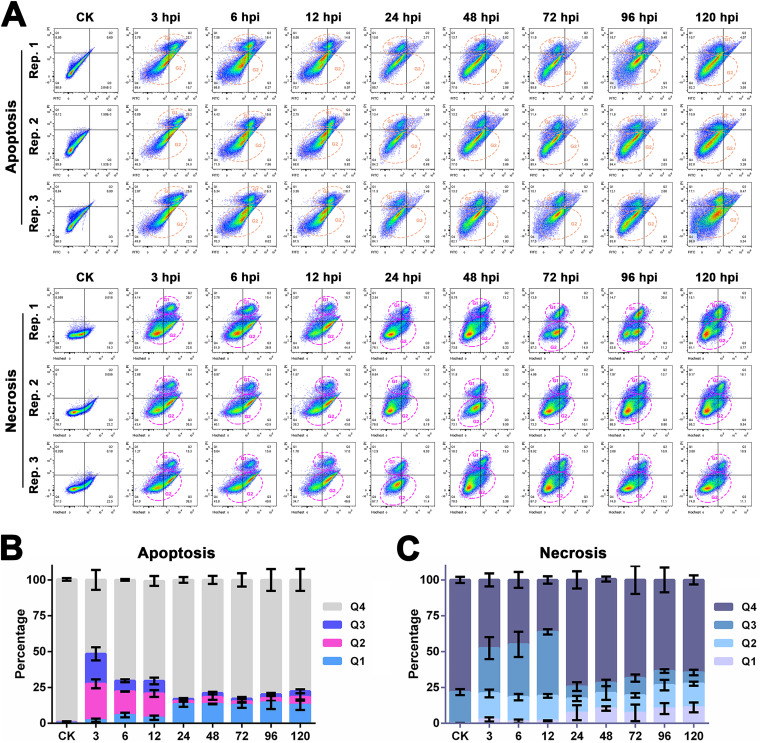
Flow cytometry (FCM) assays of HvAV-3h-infected, *in vitro*-cultured SeFB cells. (A) Two different fluorescent staining methods were employed to detect apoptosis (annexin V-FITC/PI staining, top) or necrosis (Hoechst/PI staining, bottom) in the HvAV-3h-infected SeFB cells. Three replicates were performed. The major cell groups are marked by dashed circles and designated G1 and G2. (B) The cells stained with annexin V-FITC and PI were statistically analyzed. (C) The cells stained with Hoechst stain and PI were statistically analyzed. Mean values ± standard errors are shown.

In our previous study, infection with HvAV-3h was not followed by typical apoptosis in the host larval fat bodies and hemocytes of Spodoptera exigua or Helicoverpa armigera larvae (19). That is, the observed cells did not produce typical apoptotic bodies but, instead, contained vesicles and an intact nuclear structure (19). To further confirm the biochemical characteristics of HvAV-3h-infected cells that cannot be observed by transmission electron microscopy, flow cytometry (FCM) assays were employed to detect the RCD characteristics of *in vitro*-cultured S. exigua fat body cells (SeFB) after infection with HvAV-3h (Fig. 1). After staining with fluorescein isothiocyanate-conjugated annexin V (annexin V-FITC) and propidium iodide (PI), only one cell group was detected in the healthy SeFB cells (used as control, recorded as CK SeFB cells), and >99% of CK SeFB cells were in quadrant 4 (Q4) with low PI and FITC intensities, indicating that 99% of CK SeFB cells were not apoptotic (Fig. 1A, top). After infection with HvAV-3h, cells were separated into two groups (marked with orange dashed circles in Fig. 1A). From 3 to 12 h postinfection (hpi), the cells in G_1_ phase seemed to move from Q2 to Q1 and finally stayed in Q1 from 48 to 120 hpi. Further statistical analysis indicated that the proportion of annexin V-FITC-binding positive but PI-negative cells (distributed in Q3; commonly annotated as early apoptotic cells) increased significantly after infection with HvAV-3h to approximately 21% at 3 hpi and was 7% to 9% at 6 and 12 hpi, but no more than 3% from to 24 to 120 hpi (Fig. 1B). The proportion of annexin V-FITC-binding and PI-positive cells (distributed in Q2, commonly annotated as late apoptotic cells) increased sharply at 3 hpi and remained at approximately 20% until 12 hpi, while the proportion of Q2 cells decreased to no more than 5% from 24 to 120 hpi. It is worth noting that the proportion of cells distributed in Q1 (PI-positive but annexin V-FITC-binding-negative cells, commonly annotated as dead cells) was low at 3 to 12 hpi (no more than 5%) but increased to 12% to 15% at 24 to 120 hpi. After staining with Hoechst stain and PI, only one cell group was detected in the CK SeFB cells, but it was divided into two groups after infection with HvAV-3h, and three groups of cells could be distinguished after 72 hpi (Fig. 1A, bottom, marked with pink dashed circles). Similar to the FCM results described above, the cell membrane became permeable from to 3 to 12 hpi but recovered in the following infection stages (Fig. 1C).

### An antiapoptosis phenotype was found in HvAV-3h-infected SeFB cells.

**FIG 2:**
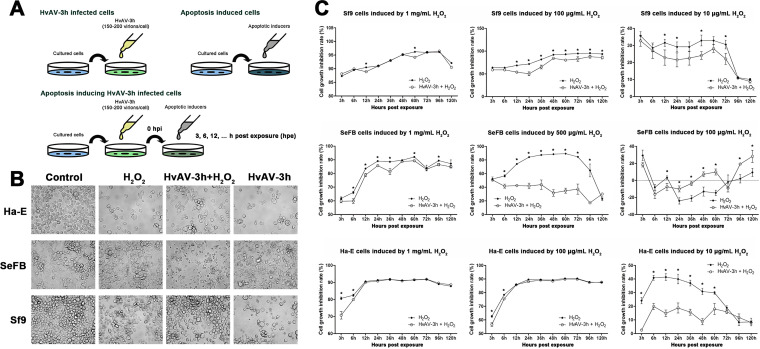
Effects of hydrogen peroxide (H_2_O_2_) on HvAV-3h-infected cells. (A) Schematic diagram of cellular treatments. The cultured SeFB, Ha-E, or Sf9 cells were inoculated with HvAV-3h-containing cell culture medium or healthy hemolymph-containing cell culture medium (control). The control cells or the HvAV-3h-infected cells (0 hpi) were then exposed to H_2_O_2_, and the experiments were performed at the indicated times postexposure. (B) The morphologies of control cells or HvAV-3h-infected cells, as well as the cells exposed to H_2_O_2_, at 48 hpe. (C) Cell growth inhibition rates of healthy SeFB cells or HvAV-3h-infected SeFB cells exposed to H_2_O_2_ (mean values ± standard errors). Asterisks indicate statistical differences between the means of the cell growth inhibition rates of H_2_O_2_-treated healthy SeFB cells or HvAV-3h-infected SeFB cells at each tested time based on the independent *t* test (α = 0.05).

Ascovirus had been considered an insect virus that used host cell apoptosis (16). Although the HvAV-3h-infected SeFB cells exhibited a permeable cell membrane in the early stage (3 to 12 hpi), which was not in agreement with the biochemical characteristics of apoptosis (Fig. 1), it was still necessary to confirm the possible relationship of ascovirus infection and host cell apoptosis. Thus, hydrogen peroxide (H_2_O_2_) was employed as an apoptosis inducer to test the difference in tolerance of the HvAV-3h-inoculated cells (HvAV-3h-infected and H_2_O_2_-treated cells [HvAV+H_2_O_2_]) and healthy S. exigua larval hemolymph-inoculated cells (H_2_O_2_-treated cells) (Fig. 2). Both the cellular morphological changes and cell viability were detected to test whether the HvAV-3h-inoculated cells had a stronger tolerance of H_2_O_2_-induced apoptosis than the control cells. A diagram of cells under different treatments is provided in Fig. 2A. After inoculation with HvAV-3h for 48 h, aggregation, swelling, or detachment from the bottom of the cell culture disks was observed in all tested cell lines (Ha-E, SeFB, and Sf9) (Fig. 2B). Compared to control cells, most of the cells exposed to H_2_O_2_ were detached from the bottoms of the cell culture disks, and swollen and malformed cells can be seen in the figure, especially in the H_2_O_2_-treated SeFB and Sf9 cells. The morphology of H_2_O_2_-treated HvAV-3h-infected cells was different from that of HvAV-3h-infected cells or H_2_O_2_-treated cells. Fewer cells were detached from the bottoms of the cell culture disks, and fewer malformed cells were found.

Cell viability was determined by MTT [3-(4,5-dimethyl-2-thiazolyl)-2,5-diphenyl tetrazolium bromide] staining. Compared to control cells, infection with HvAV-3h or H_2_O_2_ treatment inhibited cell viability (data not shown), and the cell growth inhibition rate was calculated from the cell viability of the treated cells compared to that of the control cells (Fig. 2C). Based on general linear model (GLM) analysis, the cell growth inhibition rate of H_2_O_2_-treated HvAV-3h-infected Sf9 cells was significantly lower than that of H_2_O_2_-treated control Sf9 cells for both 100-μg/mL and 10-μg/mL H_2_O_2_-treated cells (all *P < *0.05). Significant differences between the H_2_O_2_-treated, HvAV-3h-infected cells and treated Ha-E control cells were only found for cells treated with 100 μg/mL H_2_O_2_ (*P < *0.05). For the SeFB cells, significant differences were found between the 500-μg/mL H_2_O_2_-treated HvAV-3h-infected cells and the treated control cells (*P < *0.05). These results indicate that infection with HvAV-3h helps the host cell survive H_2_O_2_-induced cell apoptosis.

### Changes in cell membrane permeability against chemical apoptosis inducers were detected in HvAV-3h-infected cells.

**FIG 3:**
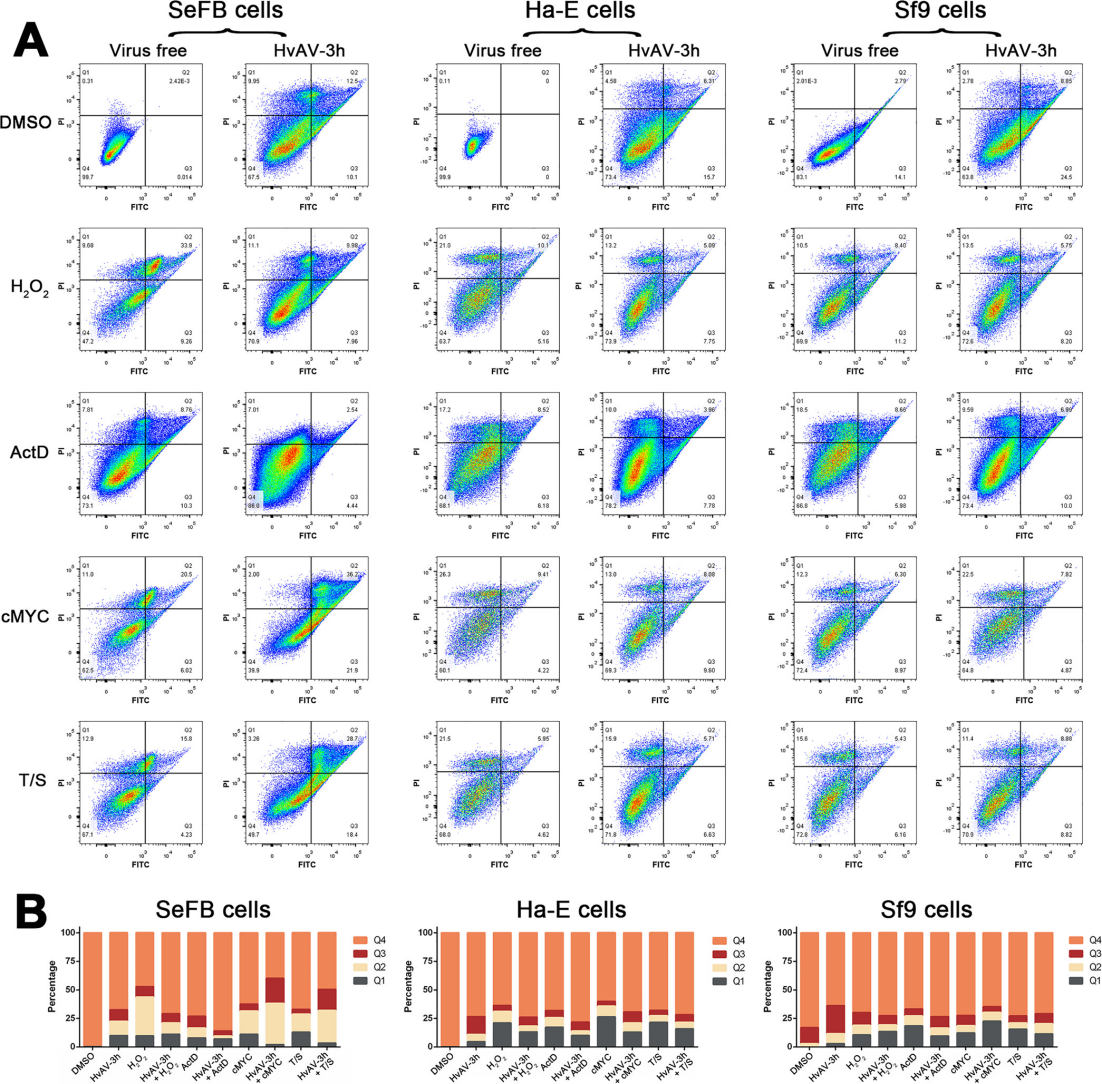
FCM analysis of virus-infected or uninfected cells exposed to different apoptosis inducers. (A) SeFB, Ha-E, or Sf9 cells were inoculated with HvAV-3h-containing medium (HvAV-3h) or healthy larval-hemolymph-containing medium (virus free), and at 24 h postinfection, the cells were exposed to 100 μg/mL H_2_O_2_, 5 μg/mL actinomycin D (ActD), 5 μg/mL cMYC inhibitor, 4 μg/mL TNF-α plus SM-164 apoptosis inducers (T/S), or 5 μg/mL dimethyl sulfoxide (DMSO). After 24 h of exposure, the cells were collected and stained with annexin V-FITC and PI, followed by FCM assays. (B) Histograms of the proportions of cells distributed in different quadrants after FCM assays.

In order to test the possible ability of HvAV-3h-infected cells to resist chemical-induced apoptosis, three different insect cell lines (SeFB, Ha-E, and Sf9) were inoculated with healthy larval hemolymph (virus free) or HvAV-3h-containing medium (HvAV-3h) for 24 h, followed by induction with dimethyl sulfoxide (DMSO; used as a control), H_2_O_2_, actinomycin D (ActD), cMYC inhibitor, or tumor necrosis factor alpha (TNF-α) plus SM-164 apoptosis inducers (T/S) for another 48 h (Fig. 3). The treated cells were collected and stained with FITC-conjugated annexin V and PI, and FCM was employed to detect the biochemical characteristics of the treated cells (Fig. 3A). Only one cell group was detected from the non-inducer-treated, virus-free SeFB or HaFB cells, and >99% of these cells were in quadrant 4 (Q4), of low PI intensity and low FITC intensity, indicating that 99% of the healthy SeFB or Ha-E cells had low membrane permeability. None of the inducers added to virus-free Sf9 cells resulted in only one cell group, but about 17% of the cells were FITC positive. After the addition of H_2_O_2_, cMYC inhibitor, or T/S, the virus-free cells were separated into two cell groups for the three insect species cells tested: one group was mainly distributed in Q4, and the other was mainly distributed in the high-PI-intensity quadrants (Q1-Q2). The H_2_O_2_, cMYC inhibitor, or T/S-induced HvAV-3h-infected cells also yielded two cell groups, but these groups were closer than the two cell groups for the chemically induced virus-free cells. The ActD-induced cell groups were not obviously separate from those of the other three inducer-treated cells. Among the three insect species cells tested, Ha-E and Sf9 cells showed similar results after different treatments, whereas the SeFB cells had more differentiated results. In the SeFB cells, the percentages of annexin V-FITC-binding and PI-positive cells (Q2) for the H_2_O_2_- or ActD-treated HvAV-3h-infected cells (Fig. 3B) were decreased compared to the percentages for the H_2_O_2_- or ActD-treated virus-free cells, whereas the proportions of Q2 cells of cMYC inhibitor- or T/S-treated HvAV-3h-infected cells were increased compared to the proportions of Q2 cells of cMYC inhibitor- or T/S-treated virus-free cells (Fig. 3B). The annexin V-FITC-binding positive but PI-negative cells (Q3) were decreased in cMYC inhibitor- or T/S-treated HvAV-3h-infected cells compared to their proportions in cMYC inhibitor- or T/S-treated virus-free cells (Fig. 3B). Similar results were found in the treated Ha-E or Sf9 cells, but the changes were not obvious. These results indicated that the cell membrane permeability of host cells treated with apoptosis inducers can be changed by infection with HvAV-3h, which can decrease H_2_O_2_- or ActD-induced cell membrane permeability but increases cMYC inhibitor- or T/S-induced cell membrane permeability.

### Viral DNA replication was altered after apoptosis inducers were added.

**FIG 4:**
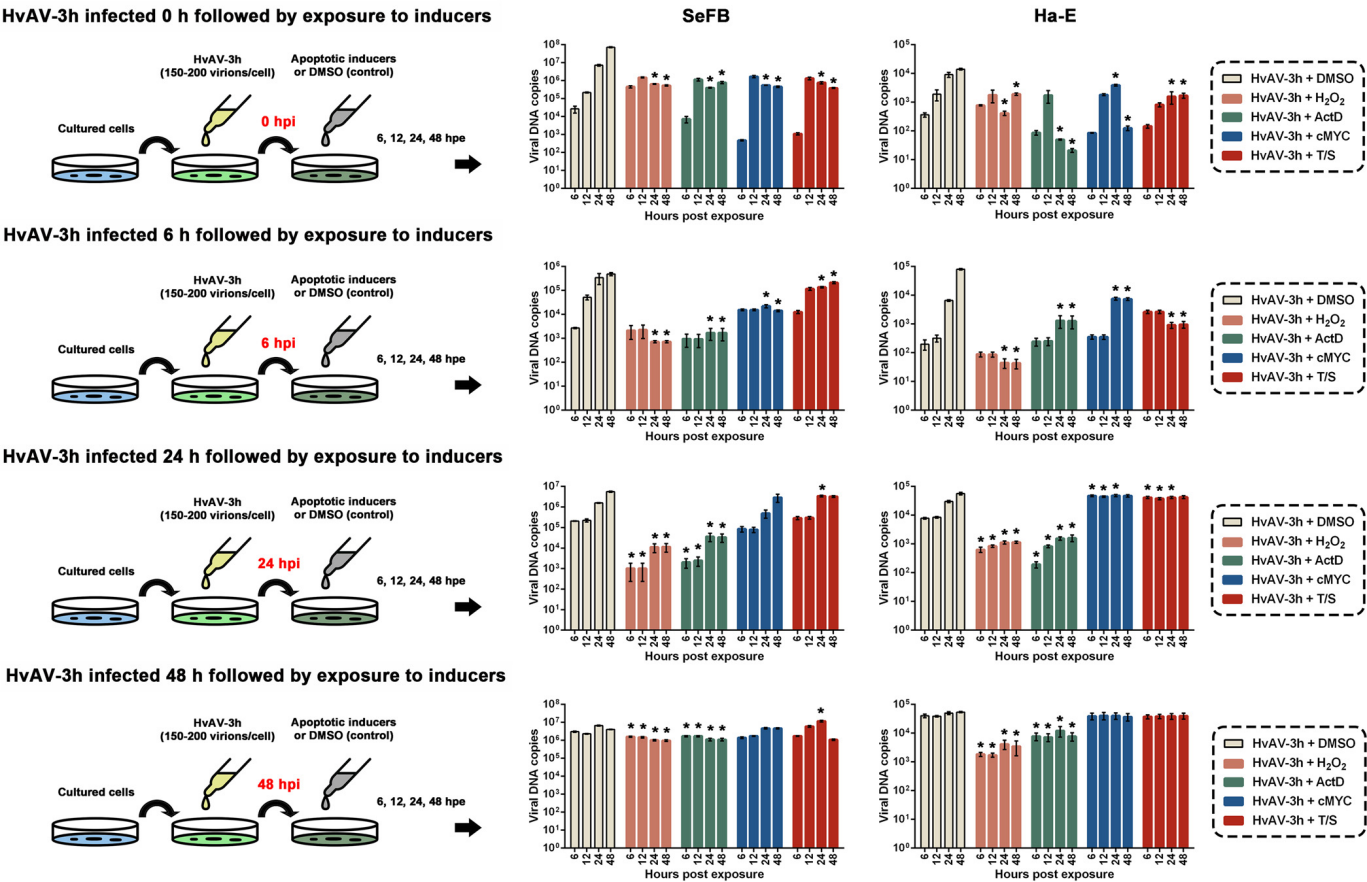
Effects of induced apoptosis on viral DNA replication. SeFB and Ha-E cells were inoculated with HvAV-3h. At 0, 6, 24, and 48 hpi, infected cells were exposed to 100 μg/mL H_2_O_2_, 5 μg/mL ActD, 5 μg/mL cMYC inhibitor, 4 μg/mL T/S, or 5 μg/mL DMSO. At 6, 12, 24, and 48 hpe, cells were collected and viral DNA copies were determined. Mean values ± standard errors are shown. Asterisks indicate statistical differences in viral DNA copies between cells exposed to apoptosis inducers and those exposed to DMSO (control) at each tested time point based on one-way ANOVA (α = 0.05).

From the results described above, we can see that the biochemical characteristics of HvAV-3h-infected cells were different at different infection stages (Fig. 1) and the responses of HvAV-3h-infected cells to different apoptosis inducers were different (Fig. 3), but the effects of the induced apoptosis on the ascoviral-DNA replication were not determined. Thus, the apoptosis inducers were added to the HvAV-3h-infected cells at different infectious stages to test their effects on the viral DNA replication. SeFB, Ha-E, or Sf9 cells were inoculated with HvAV-3h, and at 0, 6, 24, and 48 hpi, DMSO (used as a control), H_2_O_2_, ActD, cMYC inhibitor, or TNF-α plus SM-164 apoptosis inducers (T/S) were added to the HvAV-3h-infected cells. Subsequently, cells were collected and viral DNA copies were determined (Fig. 4). At 0 hpi, the viral DNA copies varied after the apoptosis inducers were added, but no significant differences were found between the viral DNA copies of the infected cells exposed to DMSO and those exposed to inducers at 6 and 12 h postexposure (hpe) for both SeFB and Ha-E cells (all *P > *0.05). Significant decreases in viral DNA copy number were found in the apoptosis inducer-treated cells compared to those in the DMSO-treated cells at 24 and 48 hpe (all *P < *0.05). Similar results were found in the apoptosis inducer-treated HvAV-3h-infected SeFB cells and the HvAV-3h-infected Ha-E cells at 12 hpi, where the viral DNA contents were not significantly decreased at 6 and 12 hpe (all *P > *0.05) but were significantly decreased at 24 and 48 hpe (all *P < *0.05). At 24 hpi, HvAV-3h-infected cells exposed to H_2_O_2_ or ActD invariably had decreased viral DNA copies compared to the numbers in the cells exposed to DMSO at each tested time point for both insect species cells tested (all *P > *0.05). In contrast, significantly increased viral DNA copies were found in the T/S-treated HvAV-3h-infected SeFB cells (24 hpe) and cMYC inhibitor- or T/S-treated HvAV-3h-infected Ha-E cells (6, 12, and 24 hpe). At 48 hpi, decreased viral DNA copies were found in H_2_O_2_- or ActD-treated SeFB and Ha-E cells (all *P > *0.05). Significantly increased viral DNA copies were found in cMYC inhibitor- or T/S-treated HvAV-3h-infected SeFB cells (24 hpe) (all *P < *0.05). HvAV-3h in the Sf9 cells always produced low viral DNA copies, which indicates that Sf9 cells might not be suitable for HvAV-3h infection, although after exposure to apoptosis inducers, viral DNA replication was inhibited (Fig. S1 in the supplemental material). These results indicated that the infection process of HvAV-3h in Sf9 cells was different from those in SeFB or Ha-E cells. In addition, the effects of H_2_O_2_ and ActD on the infection processes of HvAV-3h were different from those of cMYC inhibitor and T/S, which is in agreement with the FCM results shown in Fig. 3.

10.1128/mbio.03119-22.1 FIG S1 Viral DNA replication in the Sf9 cells to which chemical inducers were added. Download FIG S1, PDF file, 0.1 MB. Copyright © 2023 Yu et al. 2023 Yu et al. https://creativecommons.org/licenses/by/4.0/ This content is distributed under the terms of the Creative Commons Attribution 4.0 International license.

### Effects of apoptosis inducers on the expression of SeCasps and SeIAP.

**FIG 5:**
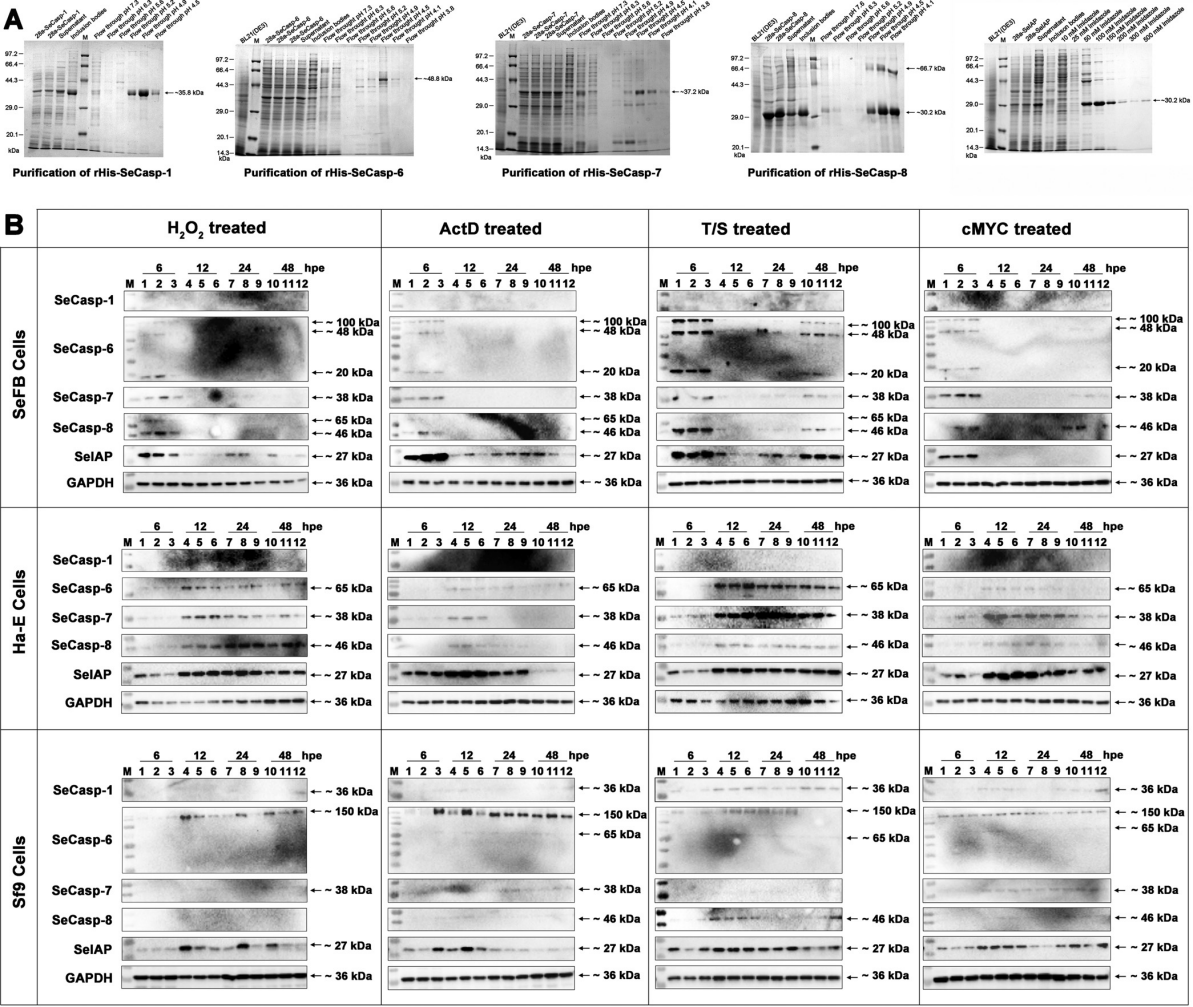
Preparation of antiserum against Spodoptera exigua caspases (SeCasps) and confirmation of the expression of SeCasps in apoptosis-induced cells. (A) Prokaryotic expression and protein purification of four SeCasps and an S. exigua inhibitor of apoptosis protein (SeIAP). The purified proteins were used as antigens to prepare polyclonal antisera. (B) Western blot assays of the expression of SeCasps and SeIAP in apoptosis-induced SeFB, Ha-E, and Sf9 cells.

In order to further study the biochemical characteristics of HvAV-3h-infected cells, Spodoptera exigua-encoded apoptosis-associated proteins were selected and used as antigens to prepare polyclonal antisera for further use. Four Spodoptera exigua caspase genes (*SeCasp*s) and an S. exigua inhibitor of apoptosis protein gene (*Seiap*) were expressed and purified (Fig. 5A), and the purified proteins were used as antigens to produce polyclonal antisera. The prepared antisera were used to detect changes in the expression of specific proteins in SeFB, Ha-E, and Sf9 cells after induction with different chemicals (Fig. 5B). Through specific detection with glyceraldehyde-3-phosphate dehydrogenase (GAPDH) antiserum, an immune band of approximately 36 kDa was detected in the protein samples of each cell, indicating that the protein samples were well prepared. In the tested SeFB cell samples, no clear bands were detected by SeCasp-1 antiserum, but clear bands were detected with SeCasp-6, SeCasp-7, SeCasp-8, and SeIAP antisera, and most of these were in the samples collected at 6 h postexposure. Cleavage was detected in the expressed SeCasp-6, in which approximately 100-kDa, 48-kDa, and 20-kDa bands were detected. In the treated Ha-E cell samples, no clear bands were detected by SeCasp-1 antiserum and no cleavage bands were detected for any of the four tested SeCasps after the addition of different apoptosis inducers, and yet, clear bands for the four SeCasps were observed in the samples collected at 12 h to 48 h postexposure. In the tested Sf9 cells, weak bands were detected for the T/S-treated cells using SeCasp-1 antiserum, an approximately 150-kDa band was detected with SeCasp-6 antiserum, which was different from the bands detected in the SeFB or Ha-E cells, and almost no clear bands were detected with SeCasp-7 or SeCasp-8 antisera. Clear bands with a molecular weight of approximately 27 kDa were detected with SeIAP antiserum, but no obvious differences were found among the different apoptosis inducer treatments.

### Comparison of the biochemical characteristics of HvAV-3h-infected SeFB cells and Ha-E cells.

**FIG 6:**
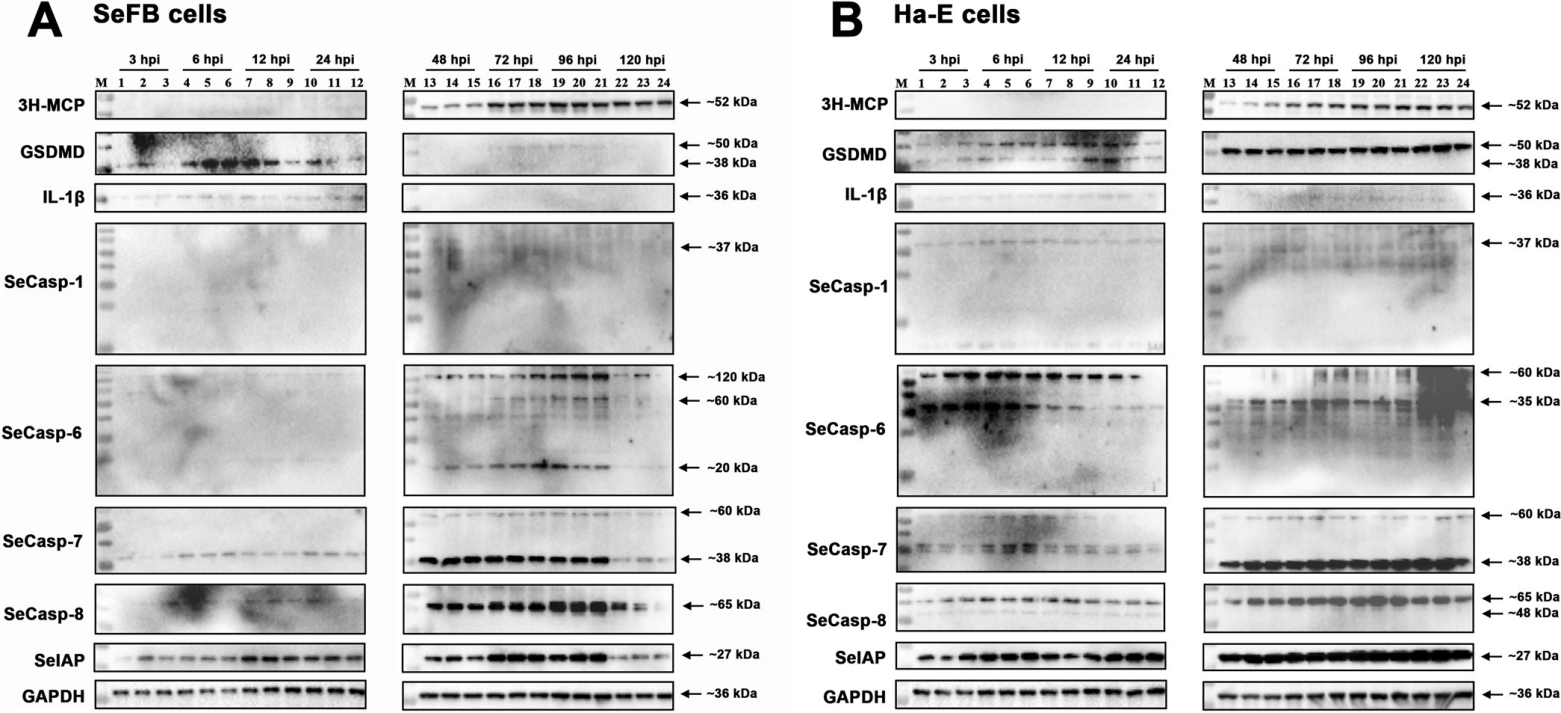
Western blot assays of the expression of regulated cell death (RCD)-associated proteins in HvAV-3h-infected SeFB or Ha-E cells. At each time point, three protein samples were collected from each HvAV-3h-infected cell culture. GAPDH antiserum and MCP antiserum were used as reference antibodies to detect protein sample preparation and HvAV-3h infection, respectively. SeCasps and SeIAP antisera prepared in this study were used to detect apoptosis-associated protein expression. Commercially obtained human GSDMD and human IL-1β polyclonal antibodies were used to identify the possible RCD types in HvAV-3h-infected cells.

To clarify the possible regulated cell death (RCD) types in host cells, antibodies against gasdermin D (GSDMD) and interleukin-1β (IL-1β) were used to detect the cleavage of GSDMD or release of IL-1β inflammatory factors during the infection processes of HvAV-3h. Through specific detection with GAPDH antiserum, an immune band of approximately 36 kDa was detected in the protein samples of each cell type, indicating that the protein samples were well prepared (Fig. 6). The results showed that in both Ha-E and SeFB cells, major capsid protein (MCP) was expressed at 48 hpi and continued to be expressed until 120 hpi. In Ha-E cells infected with HvAV-3h, GSDMD showed two prominent bands at approximately 38 kDa and 50 kDa at 3 to 24 hpi. However, only bands of approximately 50 kDa remained at 48 to 120 hpi. GSDMD in the HvAV-3h-infected SeFB cells was different from that in the infected Ha-E cells, which had an ~38-kDa immune band at 3 to 24 hpi and an ~50-kDa immune band at 48 to 120 hpi. No clear immune bands were detected with the IL-1β antibody in any HvAV-3h-infected Ha-E cells or HvAV-3h-infected SeFB cells (3 to 120 hpi). SeCasp-1 was not detected in HvAV-3h-infected Ha-E or SeFB cells. The expression pattern of SeCasp-6 in HvAV-3h-infected Ha-E cells was consistent from 3 to 120 hpi, containing two prominent bands (approximately 60 kDa and 35 kDa, respectively), while in the HvAV-3h-infected SeFB cells, no clear bands were detected in the 3- to 24-hpi protein samples. However, three prominent bands (approximately 120 kDa, 60 kDa, and 20 kDa) were detected in the 48- to 120-hpi protein samples. The expression pattern of SeCasp-7 was almost the same in the two tested cell species: a weak ~60-kDa band was consistent from 3 to 120 hpi, and an ~38-kDa band was weakly detected from 3 to 24 hpi but was clearly detected at 48 to 120 hpi. A prominent ~65-kDa band was detected in the HvAV-3h-infected Ha-E and SeFB cells. This band was weak in the 3- to 24-hpi samples but thickened in the 48- to 120-hpi samples. SeIAP was detected in all of the tested HvAV-3h-infected cells, and compared to the expression patterns of the four tested SeCasps, the expression pattern of SeIAP was not distinct between the early infection stage (3 to 24 hpi) and the late infection stage (48 to 120 hpi). These results indicate that in the early infection stage of HvAV-3h (3 to 24 hpi), host cells were more likely to undergo pyroptosis, whereas in the late infection stage (48 to 120 hpi), host cells underwent apoptosis.

### The T/S inducers promote the expression of MCP and the cleavage of SeCasp-6.

**FIG 7:**
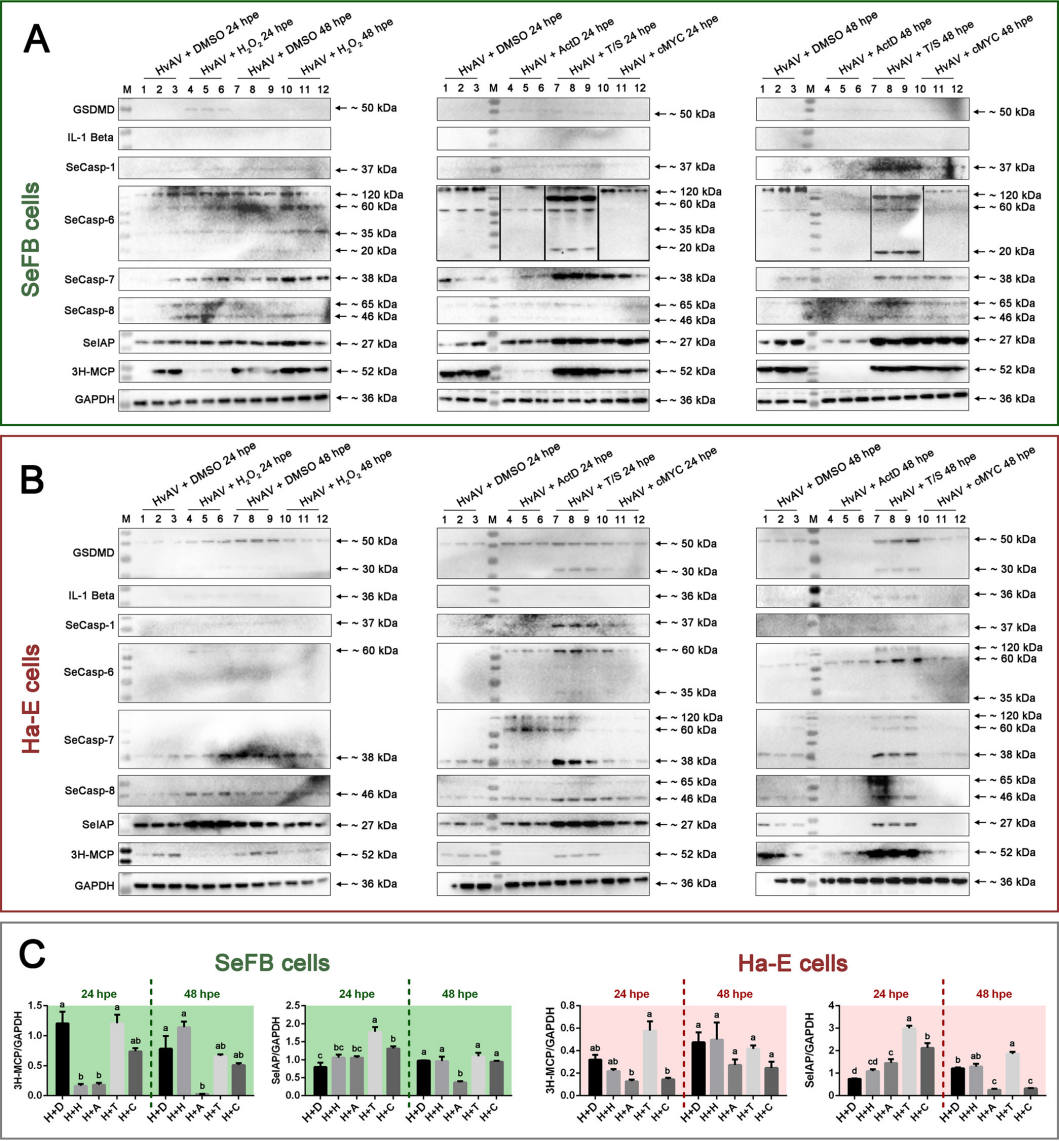
Expression of RCD-associated proteins in HvAV-3h-infected cells exposed to apoptosis inducers. (A) Expression of RCD-associated proteins in HvAV-3h-infected SeFB cells exposed to apoptosis inducers. (B) Expression of RCD-associated proteins in HvAV-3h-infected Ha-E cells exposed to apoptosis inducers. SeFB and Ha-E cells were inoculated with HvAV-3h. At 24 hpi, the infected cells were exposed to 100 μg/mL H_2_O_2_, 5 μg/mL ActD, 5 μg/mL cMYC inhibitor, 4 μg/mL T/S, or 5 μg/mL DMSO. At 24 and 48 h, the cells were collected for immunoblotting. The uncut original immunoblotting images are provided in Fig. S2. (C) Grayscale analysis of the relative expression levels of 3H-MCP and SeIAP in different treated cells. Mean values ± standard errors are shown. Different lowercase letters indicate statistical differences between the means of relative protein expression levels at each tested time based on one-way ANOVA followed by least significant difference comparisons (α = 0.05).

The inducers had different effects on different HvAV-3h-infected cells, both on the host cell membrane permeability (Fig. 3) and on the viral DNA replication ability in the host cells (Fig. 4). In order to detect their effects on the host cellular apoptosis-associated protein expression, so as to further understand the possible mechanism of ascovirus and host cell interaction on the protein level, the following experiments were performed. SeFB or Ha-E cells were infected with HvAV-3h, and at 24 hpi, chemical inducers were added to the cells. At 24 h and 48 h, cell samples were collected and used to analyze the expression of different RCD-associated proteins (Fig. 7). In the tested SeFB samples (Fig. 7A), slight GSDMD bands (~50 kDa) were detected in the HvAV-plus-inducer (HvAV+inducer) samples, whereas no bands were detected in the HvAV+DMSO samples. No immune bands were detected with the IL-1β antibody. Weak immune bands were detected with the prepared SeCasp-1 and SeCasp-8 antisera, but no obvious differences were found between the different samples. With detection by SeCasp-6 antiserum, four prominent bands (approximately 120 kDa, 60 kDa, 35 kDa, and 20 kDa) were detected in the HvAV+DMSO and HvAV+H_2_O_2_ samples, in which the ~120-kDa band was more obvious than the other three bands in each lane. This ~120-kDa SeCasp-6 band was not detected in the HvAV+ActD samples at either 24 hpe or 48 hpe. Only an approximately 120-kDa SeCasp-6 band was detected in the HvAV+cMYC inhibitor samples at both 24 hpe and 48 hpe. A clear ~20-kDa cleaved SeCasp-6 band was detected in the HvAV+T/S samples, in addition to an approximately 100-kDa band found in the HvAV+T/S samples. Only an ~38-kDa band was detected with SeCasp-7 antiserum, and the HvAV+T/S samples (24 hpe) had more obvious immune reactions than the other samples. An ~27-kDa SeIAP band was detected in all samples, and stronger immune reaction bands were detected in the HvAV+cMYC inhibitor and HvAV+T/S samples at both 24 and 48 hpe. An approximately 52-kDa immune band was detected in all tested samples with 3H-MCP antiserum. These bands were weak in the HvAV+ActD samples (at both 24 and 48 hpe) but extremely strong in the HvAV+T/S samples (at 24 hpe). Further grayscale analysis of the immunoblots indicated that the HvAV+T/S samples had the highest relative expression level of 3H-MCP at 24 hpe, but no significant differences were found between the HvAV+T/S and HvAV+DMSO samples (48 hpe, *F* = 39.31, df. = 4, 8, *P < *0.0001). In contrast, the HvAV+T/S samples had the highest relative expression level of SeIAP at 24 hpe, which was significantly higher than that of the other samples (*F* = 15.47, df. = 4, 8, *P = *0.0008) (Fig. 7C).

10.1128/mbio.03119-22.2 FIG S2 The uncut immunoblotting images from the experiment whose results are shown in Fig. 7A. Download FIG S2, PDF file, 0.1 MB. Copyright © 2023 Yu et al. 2023 Yu et al. https://creativecommons.org/licenses/by/4.0/ This content is distributed under the terms of the Creative Commons Attribution 4.0 International license.

In the tested Ha-E samples (Fig. 7B), two prominent bands of approximately 30 kDa and 50 kDa were detected with the GSDMD antibody, and the cleavage of GSDMD was clearer in the HvAV+T/S samples at both 24 and 48 hpe. Weak immune bands were detected in the HvAV+H_2_O_2_ (24 hpi), HvAV+DMSO (48 hpe), and HvAV+T/S (24 hpe and 48 hpe) samples with IL-1β antibody. Among all of the tested protein samples, only the HvAV+T/S (24 hpi) samples yielded the ~37-kDa band of SeCasp-1. An approximately 60-kDa band was detected in the HvAV+ActD, HvAV+T/S, and HvAV+cMYC inhibitor samples (at both 24 hpe and 48 hpe) with the prepared SeCasp-6 antiserum, among which the HvAV+T/S samples had the strongest immune reaction. These samples had an ~35-kDa cleaved band (at both 24 hpe and 48 hpe) and an additional band of ~120-kDa (at 48 hpe). With detection by SeCasp-7 antiserum, three prominent immune bands were detected (approximately 120 kDa, 60 kDa, and 38 kDa) in the HvAV+ActD, HvAV+T/S, and HvAV+cMYC inhibitor samples (at both 24 and 48 hpe). Among all of the samples, the HvAV+T/S samples had the strongest 38-kDa immune band using the reaction of the SeCasp-7 antiserum. SeCasp-8 showed two prominent bands, approximately 46 kDa and 65 kDa, in the HvAV+T/S and HvAV+cMYC inhibitor samples (at 24 hpe), respectively. However, only bands of approximately 46 kDa were detected in the HvAV+DMSO and HvAV+H_2_O_2_ samples. An ~27-kDa SeIAP band was detected in all samples, and stronger immune reaction bands were detected in the HvAV+H_2_O_2_ (at 24 hpe) and HvAV+T/S (at both 24 hpe and 48 hpe) samples. An approximately 52-kDa immune band was detected with 3H-MCP antiserum, and these bands were weak in the HvAV+H_2_O_2_, HvAV+ActD, and HvAV+cMYC inhibitor samples (at both 24 and 48 hpe). Further grayscale analysis of the immunoblots indicated that the HvAV+T/S samples had the highest relative expression level of 3H-MCP at 24 hpe, which was significantly higher than that of the other samples (all *P < *0.05) (Fig. 6C). In addition, the HvAV+T/S samples had the highest relative expression level of SeIAP at both 24 and 48 hpe. Significant differences were found between the HvAV+T/S samples and other samples at each tested time point (all *P < *0.05).

### Viral DNA replication is dependent on SeCasp activity.

**FIG 8:**
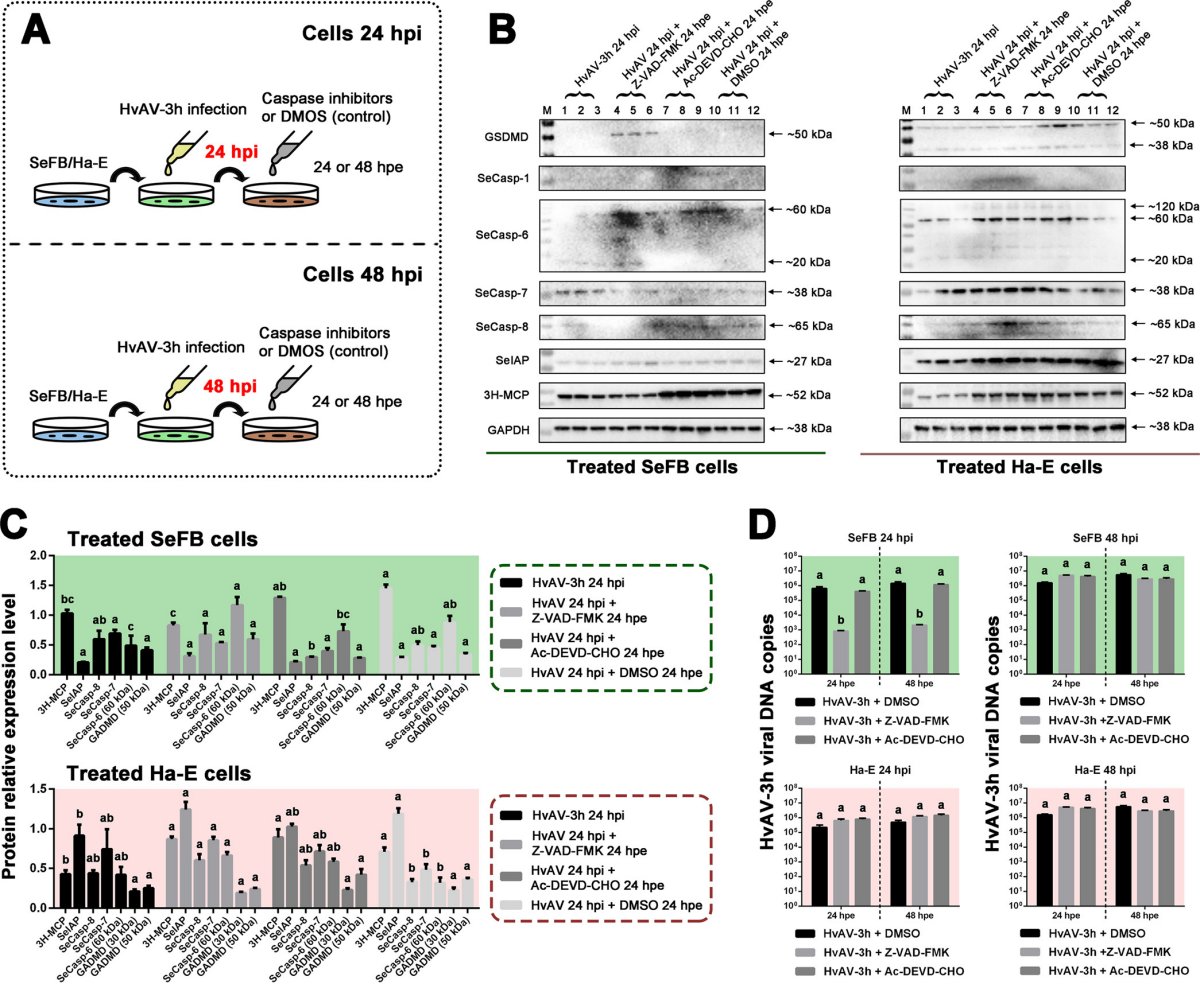
Effects of caspase inhibitors on HvAV-3h-infected cells. (A) Schematic diagram of cellular treatments. Cultured SeFB or Ha-E cells were inoculated with HvAV-3h. At 24 hpi or 48 hpi, the caspase inhibitor (Z-VAD-FMK) or the caspase-3 inhibitor (Ac-DEVD-CHO) was added to the infected cells. At 24 hpe or 48 hpe, the cells were collected to perform the following detection assays. (B) Western blot assays of RCD-associated protein expression in the HvAV-3h-infected cells exposed to caspase inhibitors. (C) Grayscale analysis of the relative expression levels of RCD-associated proteins in the different treated cells. Mean values ± standard errors are shown. Different lowercase letters indicate statistical differences between the means of protein relative expression levels at each tested time based on one-way ANOVA followed by least significant difference comparisons (α = 0.05). (D) Effects of induced apoptosis on the viral DNA replication of HvAV-3h in SeFB or Ha-E cells. Mean values ± standard errors are shown. Different lowercase letters indicate statistical differences between the means of viral DNA copies between different treated cells at each tested time based on one-way ANOVA followed by least significant difference comparisons (α = 0.05).

The results from the above-described experiments suggested that the ascoviral-DNA replication relies on the apoptosis of host cells (Fig. 4), and the cleavage of SeCasp-6 was associated with the upregulated expression of viral MCP protein (Fig. 7). Four SeCasps (SeCasp-1, SeCasp-6, SeCasp-7, and SeCasp-8) were revealed to have certain caspase activities, among which SeCasp-1, SeCasp-6, and SeCasp-7 were identified as having activities similar to that of human caspase-3 (20). The caspase inhibitor Z-VAD-FMK and caspase-3 inhibitor Ac-DEVD-CHO were employed to inhibit the caspase activity of HvAV-3h-infected cells, to test the relationship between the host cellular caspase activity and HvAV-3h infection. The caspase inhibitors were added to HvAV-3h-infected SeFB or Ha-E cells (24 hpi), and the cells were collected at 24 h and 48 h after exposure to these inhibitors (Fig. 8A). In SeFB cells, only bands of ~50 kDa were detected with the GSDMD antibody in the HvAV+Ac-DEVO-CHO-treated cells, but two prominent bands, approximately 38 kDa and 50 kDa, were detected in the treated Ha-E samples (Fig. 8B). No clear immune bands were detected with the IL-1β antibody or SeCasp-1 antiserum in any of the treated Ha-E or SeFB cells (data not shown). After exposure to the caspase inhibitors, cleavage of SeCasps was inhibited in both HvAV-3h-infected SeFB and Ha-E cells. Moreover, the detected immune bands of SeCasps were clearer in Ha-E cell samples than in SeFB cell samples. Weak SeIAP bands were detected in the treated SeFB samples, while strong SeIAP bands were detected in the treated Ha-E samples. However, no significant differences were found between the different treatments in SeFB or Ha-E cells as revealed by grayscale analysis (Fig. 8C). Bands of approximately 52 kDa were detected in all treated SeFB and Ha-E cells (Fig. 8B). Grayscale analysis of the immunoblot of the treated SeFB cells indicated that the addition of Ac-DEVD-CHO slightly inhibited the expression of 3H-MCP (compared to HvAV at 24 hpi plus DMSO at 24 hpe [*F* = 7.643, df. = 3, 6, *P = *0.0179]), whereas the addition of Z-VAD-FMK significantly inhibited the expression of 3H-MCP (compared to HvAV at 24 hpi plus DMSO at 24 hpe [*F* = 7.643, df. = 3, 6, *P* = 0.0179]). However, no such inhibition was found using the caspase inhibitors (Ac-DEVD-CHO or Z-VAD-FMK) added to HvAV-3h-infected Ha-E cells (24 hpi) and HvAV at 24 hpi plus DMSO at 24 hpe (Fig. 8B and C).

The above-described results indicated that the inhibited host cellular caspase activity might result in inhibition of the expression of the HvAV-3h virion structural protein (3H-MCP). Further analysis was performed to investigate whether the inhibited cellular caspase activity had any effect on viral DNA replication (Fig. 8D). In HvAV-3h-infected SeFB cells, when Ac-DEVD-CHO was added at 24 hpi, significantly decreased viral DNA copies were found at both 24 and 48 hpe (*F* = 5.266, df. = 3, 6, *P = *0.0406), but no such decrease in viral DNA copies was found when Ac-DEVD-CHO was added at 48 hpi. No significant decrease in viral DNA replication was observed in the Z-VAD-FMK-treated HvAV-3h-infected SeFB cells, regardless of whether Z-VAD-FMK was added at 24 or 48 hpi. No significant differences were found between the viral DNA copies in the caspase inhibitor-treated and DMSO-treated Ha-E samples, regardless of the addition of inhibitors or duration of cell exposure to the inhibitors.

### The interaction between SeCasps and SeIAP was inhibited by infection with HvAV-3h.

**FIG 9:**
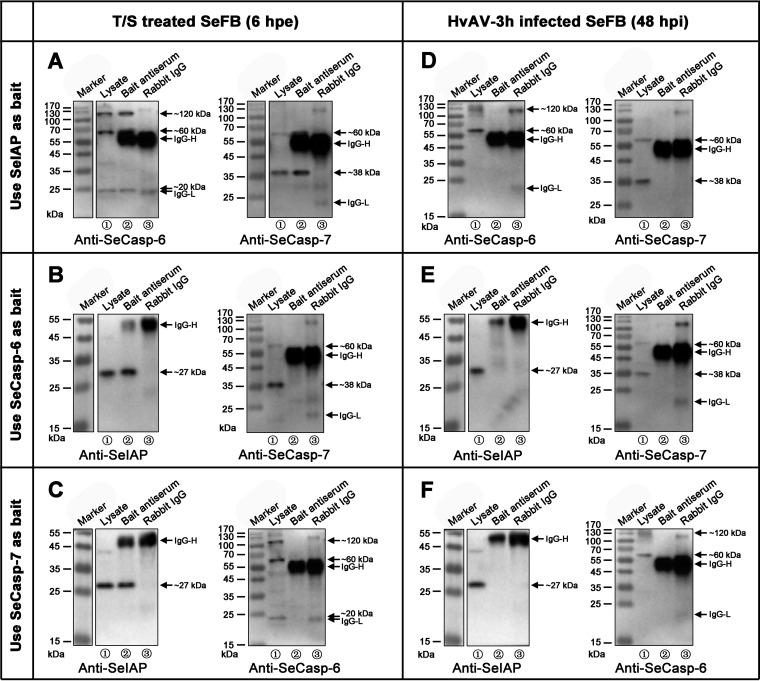
Coimmunoprecipitation assays for SeCasp-6, SeCasp-7, and SeIAP. SeFB cells were treated with 4 μg/mL TNF-α plus SM-164 apoptosis inducers (T/S) for 6 h (A to C), or infected with HvAV-3h for 48 h (D to F). The lysates of the treated cells were immunoprecipitated (IP) with antiserum against SeIAP (A and D), SeCasp-6 (B and E), or SeCasp-7 (C and F) or with rabbit immunoglobulin (IgG; used as a negative control). The samples were separated by SDS-PAGE, blotted, and probed with the indicated antibodies.

Coimmunoprecipitation assays were performed to investigate the possible interactions between SeCasp-6, SeCasp-7, and SeIAP under two different conditions (with or without HvAV-3h). As shown by the results in Fig. 8, specific protein bands that were consistent with the previously obtained results (Fig. 5 and 6) were detected in all lysates. The heavy chain (approximately 60 kDa) and light chain (approximately 20 kDa) of rabbit immunoglobulin G (IgG) were detected in the immunoprecipitated lanes, which was due to the direct binding of the secondary antibodies to rabbit IgG. Rabbit IgG did not precipitate any specific proteins, indicating that the precipitated protein bands were due to the interaction with the bait protein. In the TNF-α+SM-164 apoptosis inducer group, SeFB cell samples were coimmunoprecipitated with anti-SeIAP-precipitated SeCasp-6 and SeCasp-7 (Fig. 9A, lanes 2 and 5). Likewise, coimmunoprecipitation with anti-SeCasp-6 or SeCasp-7 precipitated SeIAP (Fig. 9B and C, respectively, lanes 2). However, neither SeCasp-7 nor SeCasp-6 precipitated during coimmunoprecipitation with anti-SeCasp-6 (Fig. 9B, lane 5) or anti-SeCasp-7 (Fig. 9C, lane 5). In the HvAV-3h-infected SeFB cell samples, no precipitated protein bands were found in the coimmunoprecipitation with bait protein antisera (Fig. 9D to F), suggesting that the interaction between SeIAP and SeCasp-6 or SeCasp-7 was blocked.

## DISCUSSION

For many years, the apoptosis response of host cells to infection by a virus and the antiapoptosis properties of viruses in the infected cells comprised the major described type of RCD regarding the interaction of insect viruses and their host cells (21–29). Autophagy is another type of RCD that has been studied in the context of the pathogenesis of insect viruses (30–32). Compared to the RCD types reported by NCCD, few are known in insect cells. Several studies have suggested that a transformation occurs between apoptosis, necrosis, and pyroptosis (33–39). Therefore, we believe that the inhibition of apoptosis following viral infection, which has been verified by most insect virologists, might result from other types of RCD and might also transform into other types of RCD. Thus, we first investigated the possible RCD type of SeFB cells after infection with HvAV-3h at different infection stages using FCM (Fig. 1). A demarcation line was found at 24 hpi in the HvAV-3h-infected SeFB cells. Before 24 hpi, the permeability of the host cell membranes was extremely high, but after 24 hpi, the permeability of the host cell membranes decreased dramatically (Fig. 1). This early phenomenon of high cell permeability is not consistent with the biochemical characteristics of apoptosis. As frequently reported, at the early stage of apoptosis, phosphatidylserine (PS) turns from the inside to the outside of the lipid membrane, a process that can be blocked with the high-affinity protein annexin V. Therefore, annexin V is used as one of the sensitive indicators to detect the early-apoptosis stage of cells. However, in our FCM analysis, annexin V-positive and PI-positive cells were always detectable from to 3 to 12 hpi (Fig. 1), which indicates that necrosis- or pyroptosis-like RCD does occur in the early stage of HvAV-3h infection. This was consistent with the transmission electron microscopy (TEM) results in our previous studies (19). Before 24 hpi (the early stage of infection with HvAV-3h), no fat body cells or hemocytes were observed that displayed typical apoptotic characteristics, but there were hemocytes with pyroptotic characters—swollen cells with intact nuclei and degraded organelles, except for mitochondria (19).

As described above, the pathogenesis of ascoviruses has been described as an apoptosis-like process, as ascoviruses need to use apoptotic bodies produced by host cells during the process of apoptosis to package their own vesicles. To verify the relationship between ascovirus infection and host cell apoptosis, we used hydrogen peroxide to induce apoptosis of host cells after infection with an ascovirus. If the infection process of the ascovirus takes advantage of apoptosis from the host cell, the addition of hydrogen peroxide would rapidly lyse the host cell, accelerating the pathogenic process of the ascovirus, and vice versa. However, the results showed that HvAV-3h-infected cells induced by hydrogen peroxide had a reduced growth inhibition rate compared to that of healthy cells (Fig. 2). This shows that, similar to the reports on baculovirus and iridovirus (40–45), ascovirus infection actually inhibits host cell apoptosis.

In cases of an antiapoptosis-like, rather than an apoptosis-like process in the pathogenesis of ascoviruses, more studies have been performed to detect the relationship between apoptosis and the possible RCD of host cells and viral DNA replication. Apoptosis inducers (H_2_O_2_, ActD, cMYC inhibitor, and TNF-α+SM-164 [T/S]) were added to the cells at different infection stages. The results showed that H_2_O_2_ and ActD had similar functional effects on HvAV-3h-infected cells, while the cMYC inhibitor and T/S had similar functional effects on HvAV-3h-infected cells (Fig. 4). In most cases, exposure to apoptosis inducers resulted in decreased viral DNA copies. However, exposure to a cMYC inhibitor or a T/S inducer resulted in increased viral DNA copies at 24 hpi (Fig. 4). According to previous studies, H_2_O_2_ can increase the cellular degree of oxidative stress, and treated cells would undergo Fas-mediated apoptosis (46). Actinomycin D triggers cell death by affecting several different pathways, including the Jun N-terminal protein kinase (JNK)/stress-activated protein kinase (SAPK) pathway, p35 pathway, and Kv1.3 channels, among others (47). The cMYC inhibitor (10058-F4) can activate the mitochondrial pathway by downregulating the expression of Bcl-2 and upregulating the expression of Bax so as to release cytochrome *c* (48). SM-164 is a bivalent analog of second mitochondria derived activator of caspase (Smac), which can induce the degradation of cIAP-1/2 and inhibit XIAP, so as to induce TNF-α-dependent apoptosis (49, 50). From these results, we assumed that the infection of vesicular viruses depends on different types of apoptosis, and not Fas-related apoptosis, as the induction of this type of apoptosis would lead to a sharp decline in virus replication. In contrast, ascoviruses must resist mitochondrial-related apoptosis in the early stage of infection and yet utilize this type of apoptosis in the late stage of infection.

Apoptosis in insect cells is quite different from that in mammalian cells (51–53). To further detect caspase-associated apoptosis during infection with HvAV-3h, antisera of four Spodoptera exigua caspases (SeCasps) and an S. exigua inhibitor of apoptosis protein (SeIAP) were prepared (Fig. 5A). The sequences of these genes were originally obtained from the reported transcriptome data of HvAV-3h-infected S. exigua larvae (54), and the caspase activity of these four SeCasps was verified in a previous study (20). During the Western blotting assays, the immune bands from SeCasp-1 antiserum were not clear in the SeFB and Ha-E samples. Only the Sf9 samples had some recognizable bands (Fig. 5B). This might be due to the difference between the *in vivo* and *in vitro* samples, and the immune bands of SeCasp-1 that correlated with the predicted size were detectable in the S. exigua larval protein samples (data not shown). The results of Western blotting assays of HvAV-3h-infected cells also indicated 24 h as the boundary, showing two completely different immune band patterns from the samples before 24 hpi and after 48 hpi (Fig. 6), which was consistent with the FCM results (Fig. 1). Only SeCasp-6 showed cleaved bands after 48 hpi in the HvAV-3h- infected SeFB cells, while the other SeCasps were not cleaved (Fig. 6). Western blotting results showed that IL-1β was indeed detected after infection with HvAV-3h, suggesting that necrosis or pyroptosis occurred after infection with HvAV-3h. Interestingly, the IL-1β band disappeared in the SeFB cells after 48 hpi. This may indicate that cellular necrosis or pyroptosis had been suppressed or that infected cells with necrosis or pyroptosis had died, leaving only noninfected healthy cells. The former possibility is more reasonable, since MCP bands were detectable from 48 to 120 hpi. GSDMS had two obvious bands, which indicated that pyroptosis occurred from 3 to 24 hpi and was inhibited in the subsequent infection stages. Complex interconnections have been reported for different RCD types. For instance, after the death receptor is activated, caspase can be activated, followed by the typical caspase-8→caspase-3 apoptosis pathway (39). However, if caspase-8 activity is inhibited, necrosis occurs instead of apoptosis (39). In addition, inflammatory factors (produced as a consequence of necrosis), together with stimulation provided by pathogens, can lead to pyroptosis (55). These results indicate that pyroptosis occurred from 3 to 24 hpi but was inhibited in the subsequent infection stages and transformed into an apoptosis-antiapoptosis interaction from 48 to 120 hpi.

Further immunoblotting assays indicated that TNF-α+SM-164 (T/S)-induced HvAV-3h-infected SeFB cells had clearly cleaved bands, which were not found in the other apoptosis inducer-treated HvAV-3h-infected SeFB cells (Fig. 7A). Furthermore, 3H-MCP expression was increased in T/S- induced HvAV-3h-infected SeFB cells (Fig. 7C). T/S-treated (24 hpi) HvAV-3h-infected SeFB and Ha-E cells (24 hpi) showed increased viral DNA copies (Fig. 4). This was consistent with the Western blotting results. These results suggest that in the later infection stage of HvAV-3h, the ascovirus needs to utilize the host cell TNF-related apoptosis process and that the cleavage of SeCasp-6 is associated with the TNF-related apoptosis process. The addition of a caspase inhibitor or caspase-3 inhibitor inhibited the cleavage of SeCasp-6 in the HvAV-3h-infected SeFB cells (Fig. 8B) and inhibited viral DNA replication (Fig. 8D). SeCasp-6 is a unique caspase of S. exigua with low similarity to the reported insect caspases and has been verified to have caspase activity similar to that of human caspase-2, -3, -4, -6, -8, and -9 (20). It might also function as the initiator caspase and effector caspase during HvAV-3h infection.

The inhibition of caspase-dependent apoptosis in mammalian cells is usually achieved at the level of positive death signals that result in the activation of initiator caspases (caspase-8, -9, and -10) and the subsequent activation of downstream effector caspases (caspase-3, -6, and -7) (56–59). This process is different in insect cells. For example, in Drosophila, caspase activity is regulated primarily after activation, and cells survive because they express the IAP protein (51, 52, 60–63). The expression of SeIAP was constant in all samples, and coimmunoprecipitation (co-IP) detection revealed that SeIAP interacted with SeCasp-6 and SeCasp-7 (Fig. 9). However, this interaction was inhibited after infection with HvAV-3h (48 hpi). Thus, we assumed that some viral proteins compete with SeCasp to interact with SeIAP. This possibility still needs to be verified. Based on the above-described experimental data, we have made the following summary and speculation: in the early infection stage of HvAV-3h (within 24 hpi), the host cell membrane has strong permeability (Fig. 1) and can release inflammatory factors (Fig. 6) and cleave GSDMD (Fig. 6), indicating pyroptosis. At the same time, the expression levels of SeCasp-6 and SeCasp-7 are extremely low at this stage, suggesting that there might be some degradation mechanisms (such as ubiquitination) of the host caspase. In the late infection stage of HvAV-3h (48 hpi), the host cell has almost no pyroptosis characteristics (Fig. 1) and has activated SeCasp-6 (Fig. 6), but this protein does not interact with host SeIAP (Fig. 9). This may be a result of HvAV-3h-encoded IAPs and caspase-like proteins.

In conclusion, two different RCD types were identified in the ascovirus-infected cells—a high cell membrane permeability type (pyroptosis-like) in the early infection stage (3 to 24 hpi) and a low cell membrane permeability type (antiapoptosis) in the late infection stage (48 to 120 hpi). Furthermore, the interactions of the SeCasps and SeIAP were blocked after the infection of ascovirus (Fig. 9). Ascovirus can contain 2 to 5 *iap* genes, and whether the blocked interactions were associated with these virus IAPs or not was not identified in this study. Besides, due to the great differences between the insect apoptotic processes and mammalian apoptotic processes, our results, as representative of the interaction between the virus and the host, have obvious limitations. But they still shed light for us to understand the different RCD types that exist during the virus infection.

## MATERIALS AND METHODS

### Viruses and insect cell lines.

Heliothis virescens ascovirus 3h (HvAV-3h) was isolated in Changsha City (China) and stored at −20°C (64). Laboratory colonies of beet armyworm (Spodoptera exigua) were fed artificial diets and maintained at 27 ± 1°C in a 16-h-light/8-h-dark photoperiod incubator. To propagate the ascovirus *in vivo*, the ascovirus was first inoculated into the hemocoel of 3rd instar S. exigua larvae using a sterilized insect pin dipped into HvAV-3h-containing hemolymph (about 1.1 × 10^11^ virion copies/mL). After 7 days, larvae with a distinct pale body color (one of the typical symptoms of ascovirus infection) were exsanguinated, and ascovirus-containing hemolymphs (approximately 1.1 × 10^11^ virion copies/mL) were collected and stored at −20°C for subsequent use (65).

SeFB cells (IOZCAS-SpexII-A) derived from the fat bodies of S. exigua (66), Ha-E cells derived from the embryos of Helicoverpa armigera, and Sf9 cells derived from the ovaries of Spodoptera frugiperda female adults were used in this study. These three insect cell lines were maintained in TMN-FH insect culture medium (Sigma, St. Louis, MO, USA) with 10% fetal bovine serum (FBS; Thermo Fisher Scientific, Waltham, MA, USA) at 27°C.

### Cellular infection and flow cytometry assays.

To inoculate the cells with ascovirus, the HvAV-3h-containing hemolymph mixture (1.1 × 10^11^ virion copies/mL) was diluted 1,000-fold with FBS-free SFM or TNM-FH medium (67). The diluted medium containing ascovirus was sterilized by filtration through a 0.22-μm membrane filter (Millipore, Burlington, MA, USA). Subsequently, FBS was added at a final concentration of 10%. Cells (SeFB or Ha-E) were seeded in 6-well plates at a density of 10^6^ cells per well in 1 mL of medium and allowed to attach for 1 h. Of the prepared medium containing HvAV-3h, 1 mL was added to each well to allow the ascovirus to attach to the cells. The supernatant from each well was replaced with fresh culture medium after 1 h of incubation, and this time point was recorded as 0 hpi. Cells were collected at 3, 6, 12, 24, 48, 72, 96, and 120 hpi, and the untreated cells (CK) were collected and used as controls in subsequent assays. Cellular morphology was observed daily using an inverted microscope. To detect possible necrosis of HvAV-3h-infected cells, the cells were stained using apoptosis and necrosis assay kits (Beyotime, Shanghai, China) according to the manufacturer’s instructions. Fluorescence intensity tests were conducted using flow cytometry (D×P Athena, Cytek, Fremont, CA, USA). To detect possible apoptosis of the HvAV-3h-infected cells, cells were stained using the BD Pharmingen FITC annexin V apoptosis detection kit (BD Biosciences, Franklin Lakes, NJ, USA) according to the manufacturer’s instructions, and fluorescence intensity tests were conducted using flow cytometry.

### Cell treatments and cell viability assays.

Three different cell lines (SeFB, Ha-E, and Sf9) were seeded in 96-well plates at a density of 2 × 10^4^ cells per well in 100 μL of medium and allowed to attach for 1 h at room temperature (RT). One-hundred-microliter amounts of prepared HvAV-3h-containing medium and negative-control medium were added to the wells. To avoid repeated infection, the supernatant of each well was replaced with fresh culture medium 1 h after infection, and this time point was set as 0 hpi. Hydrogen peroxide solution (H_2_O_2_) (Jinhuada, China) was diluted with TNM-FH cell culture medium containing FBS to generate 1,000-, 500-, 100-, and 10-μg/mL dilutions. The prepared H_2_O_2_ solutions were added to HvAV-3h-infected cells or negative control cells. At 3, 6, 12, 24, 36, 48, 60, 72, 96, and 120 h postexposure (hpe), relative cell viability was determined using MTT [3-(4,5-dimethylthiazol-2-yl)-2,5-diphenyltetrazolium bromide] assays (68).

### Confirmation of the antiapoptotic ability of HvAV-3h-infected cells.

Three different cell lines (SeFB, Ha-E, and Sf9) were seeded in 6-well plates at a density of 1 × 10^6^ cells per well in 1 mL of medium and allowed to attach for 1 h at RT. One milliliter of HvAV-3h- containing medium was added to each well to perform the ascovirus infection according to the procedures described above. The control cells or HvAV-3h-infected cells were incubated with 100 μg/mL H_2_O_2_, 5 μg/mL actinomycin D (ActD; Solarbio, Beijing, China), 5 μg/mL cMYC inhibitor (10058-F4; Beyotime, Shanghai, China), or 4 μg/mL TNF-α plus SM-164 apoptosis inducers (T/S, Beyotime, Shanghai, China) at 24 hpi. The treated cells were collected at 24 hpe. Cells treated with 5 μg/mL dimethyl sulfoxide (DMSO; Beyotime, Shanghai, China) were used as controls. The collected cells were washed with phosphate-buffered saline (PBS; 0.2 M, pH 7.4) three times, followed by staining with the BD Pharmingen FITC annexin V apoptosis detection kit (BD Biosciences, Franklin Lakes, NJ, USA) according to the manufacturer’s instructions. Fluorescence intensity tests were conducted using a flow cytometer (D×P Athena; Cytek, Fremont, CA, USA).

### DNA extraction and absolute quantitative PCR.

To determine whether the induced apoptosis had any effect on the viral DNA replication of HvAV-3h, DMSO or apoptosis inducers (as described above) were added to the control cells or HvAV-3h- infected cells at 0, 6, 24, and 48 hpi. At 6, 12, 24, and 48 hpe, the cells were collected, and total cellular DNA was extracted using a *SteadyPure* universal genomic DNA extraction kit (Accurate Biology, Changsha, China). Quantitative PCR (qPCR) was performed to detect the expression level of *mcp* (major capsid protein gene) with specific primers (Table 1) according to the manufacturer’s instructions for the SYBR green premix *Pro Taq* HS qPCR kit (Accurate Biology, Changsha, China). For each time point of each treatment, three biological replicates were performed, and three technical replicates were performed for each biological replicate. An *mcp* fragment containing the pEGM-T easy vector was constructed and used as a standard to establish a curve of *mcp* copies and cycle threshold (*C_T_*) values. Viral DNA copies were calculated via the standard curves, and the differences between the viral DNA contents in different treated cells within one tested time point were analyzed by one-way analysis of variance (ANOVA) and compared using the least significant difference (LSD) method in SPSS 22.0 (SPSS, Inc., Chicago, IL, USA).

**TABLE 1:** Primers used in this study

Primer	Sequence (restriction enzyme)^*a*^	Purpose^*b*^
SeC1-F	5′-AGGATCCATGGAGAATACAGGGGAAAG-3′ (BamHI)	Amplification of the CDS of *SeCasp-1*
SeC1-R	5′-ACTCGAGTCAAAATTTCAGCAGCTTGG-3′ (XhoI)	
SeC6-F	5′-AGGATCCATGTCTTTATTACAAAACTT-3′ (BamHI)	Amplification of the CDS of *SeCasp-6*
SeC6-R	5′-ACTCGAGTTAATAGACTACGCAATATT-3′ (XhoI)	
SeC7-F	5′-AGGATCCATGCTGGACGGAAAACAAGA-3′ (BamHI)	Amplification of the CDS of *SeCasp-7*
SeC7-R	5′-ACTCGAGTTACTTCTTACCAAACACCA-3′ (XhoI)	
SeC8-F	5′-AGGATCCATGTTGTCTCTAGACTCCGT-3′ (BamHI)	Amplification of the CDS of *SeCasp-8*
SeC8-R	5′-ACTCGAGTTAGGAACTTGTAAGTAAAG-3′ (XhoI)	
SeIAP-F	5′-AGGATCCATGTGGTCGTGTTCCTTAC-3′ (BamHI)	Amplification of the CDS of *Seiap*
SeIAP-R	5′-ACTCGAGCGAGAAATATAATCGCACT-3′ (XhoI)	
qMCP-F	5′-GCGATAGATATCCGCAGGAA-3′	Detection of viral DNA copies in the qPCR
qMCP-R	5′-AAACATGTTCGATGCAGCAC-3′	

a Restriction enzyme sites are underlined.

b CDS, coding DNA sequence.

### Preparation of polyclonal antiserum.

An S. exigua inhibitor of apoptosis protein gene (*Seiap*) was amplified from the cDNA of HvAV-3h-infected S. exigua larvae according to a previously described method (54). Four S. exigua caspase genes (*SeCasp-1*, *SeCasp-6*, *SeCasp-7*, and *SeCasp-8*) reported in our previous study were amplified from cDNA using specific primers (Table 1) (67). The five genes were then subcloned into the multiple cloning sites of the pET-28a(+) vector (Novogene, Beijing, China), followed by induction of expression in Escherichia coli strain BL21(DE3). The expressed 6×His tag-fused SeCasps and SeIAP were affinity purified with cOmplete His tag purification resin (Roche, Basel, Switzerland). Protein samples collected during the purification procedures were separated, followed by sodium dodecyl sulfate-polyacrylamide gel electrophoresis (SDS-PAGE). The purified protein (with purity of >95%) was sent to the Wuhan Institute of Virology, Chinese Academy of Sciences, to prepare rabbit polyclonal antiserum.

### Protein extraction and immunoblotting assays.

To test the prepared antiserum, SeFB, Ha-E, or Sf9 cells were seeded in 6-well plates and exposed to apoptosis inducers (H_2_O_2_, ActD, cMYC inhibitor, or T/S) as described above. Treated cells were collected at 6, 12, 24, and 48 h. Total protein was extracted using radioimmunoprecipitation assay (RIPA) buffer (Solarbio, Beijing, China). The extracted protein samples were separated by 12% to 15% SDS-PAGE and transferred to a nitrocellulose membrane (Millipore, Burlington, MA, USA). The prepared polyclonal antibodies (SeCasp-1, SeCasp-6, SeCasp-7, SeCasp-8, and SeIAP; 1:3,000) were used to detect the expression of SeCasps and SeIAP under different treatments. Noctuid insect-specific GAPDH (glyceraldehyde-3-phosphate dehydrogenase) polyclonal antibody (69) was used as the reference antibody. Horseradish peroxidase (HRP)-conjugated goat anti-rabbit antibody (1:5,000) (Millipore, Burlington, MA, USA) was used as the secondary antibody. The proteins were visualized using Clarity Western ECL substrate (Bio-Rad, Hercules, CA, USA).

To determine apoptosis-associated protein expression during HvAV-3h infection, HvAV-3h-infected SeFB cells or HvAV-3h-infected Ha-E cells were collected at 3, 6, 12, 24, 48, 72, 96, and 120 hpi. Total protein was extracted and separated using SDS-PAGE as described above. After being transferred to a nitrocellulose membrane, the prepared polyclonal antibodies (SeCasp-1, SeCasp-6, SeCasp-7, SeCasp-8, and SeIAP, 1:3,000) were used to detect the expression of SeCasps and SeIAP under different treatments. A polyclonal antibody against the major capsid protein (MCP; 1:3,000) was used to detect the expression of the viral structural protein of HvAV-3h. Commercial polyclonal antibodies against human gasdermin D (GSDMD) (1:1,000, 20770-1-AP; Proteintech, Wuhan, China) and human IL-1β (1:500, 16806-1-AP; Proteintech, Wuhan, China) were used as primary antibodies to detect possible expression of GSDMD or IL-1β. A GAPDH polyclonal antibody (1:4,000) was used as the reference antibody. HRP-conjugated goat anti-rabbit antibody (1:5,000) (Millipore, Burlington, MA, USA) was used as the secondary antibody. The proteins were visualized using Clarity Western ECL substrate (Bio-Rad, Hercules, CA, USA).

### Quantitative analysis of apoptosis-associated protein expression in the induced cells.

To detect the apoptosis-associated protein expression in the apoptosis inducer-treated HvAV-3h-infected cells, DMSO or apoptosis inducers were added to the HvAV-3h-infected SeFB or Ha-E cells at 24 hpi. Following this, the treated cells were collected at 24 and 48 hpe and then used for extraction of total protein. Western blotting was then performed as described above. The grayscale value of each immune band was analyzed using ImageJ software (version 1.8.0; Bharti Airtel Ltd., New Delhi, India). The grayscale values of different immune membranes were uniform for the same grayscale samples. The relative expression levels of specific protein bands were calculated as the ratio of the uniform grayscale of a specific protein to the uniform grayscale of GAPDH. The differences in protein expression levels between the different samples were analyzed by one-way ANOVA and compared using the LSD method in SPSS 22.0 (SPSS, Inc., Chicago, IL, USA).

### Treatment with caspase inhibitors.

To detect whether viral replication was required for caspase activity, two caspase inhibitors, Z-VAD-FMK (caspase inhibitor; Beyotime, Shanghai, China) and Ac-DEVD-CHO (caspase-3 inhibitor; Sigma, St. Louis, MO, USA), were used to inhibit cellular caspase activity. SeFB or Ha-E cells were seeded in 6-well plates and infected with HvAV-3h. At 24 and 48 hpi, Z-VAD-FMK or Ac-DEVD-CHO was added to the medium to obtain final concentrations of 10 μM and 20 μM, respectively. At 24 and 48 h postexposure to caspase inhibitors, the cells were collected. Immunoblotting was performed, and the grayscale of each immune band was analyzed as described above. Quantitative PCR was performed to detect viral DNA copies under different treatments, as described above. The differences in protein expression levels or viral DNA copies between different samples were analyzed by one-way ANOVA and compared using the LSD method in SPSS 22.0.

### Co-IP.

SeFB cells were collected 6 h after exposure to T/S or 48 h after infection with HvAV-3h. Cell lysates were extracted using cell lysis buffer for Western blotting and IP (Beyotime, Shanghai, China). To perform co-IP, SeIAP, SeCasp-6, and SeCasp-7 were used to capture bait proteins. Rabbit immunoglobulin G (IgG) was used as the negative-control bait protein. Immunoprecipitation was performed using Pierce protein A/G magnetic beads (catalog number 88803; Thermo Fisher Scientific, Waltham, MA, USA). The eluates of samples from each incubation were collected for immunoassays, as described above. The antisera of SeIAP, SeCasp-6, and SeCasp-7 were used as primary antibodies to detect possible interactions between SeCasps and SeIAP.
