# Supplementary material for: Apoptosis or Antiapoptosis? Interrupted Regulated Cell Death of Host Cells by Ascovirus Infection In Vitro
Source: mBio. 2023 Feb 6;14(1):e03119-22. doi: 10.1128/mbio.03119-22 (PMC9973268; doi:10.1128/mbio.03119-22)

Supplementary Figures

Figure S1

Viral DNA replication of Heliothis virescens ascovirus 3h (HvAV-3h) in the chemical induced Sf9 cells. Sf9 cells were inoculated with HvAV-3h. At 0 hpi, infected cells were exposed to 100 µg/mL H_2O_2, 5 µg/mL ActD, 5 µg/mL cMYC, 4 µg/mL T/S, or 5 µg/mL DMSO. At 0, 3, 6, 12, 24, 48, and 72 h post exposure (hpe), cells were collected, and viral DNA copies were determined. Asterisks indicate statistical differences in viral DNA copies between cells exposed to apoptotic inducers and those exposed to DMSO (control) at each tested time point based on the one way ANOVA ($\alpha = 0.05$).

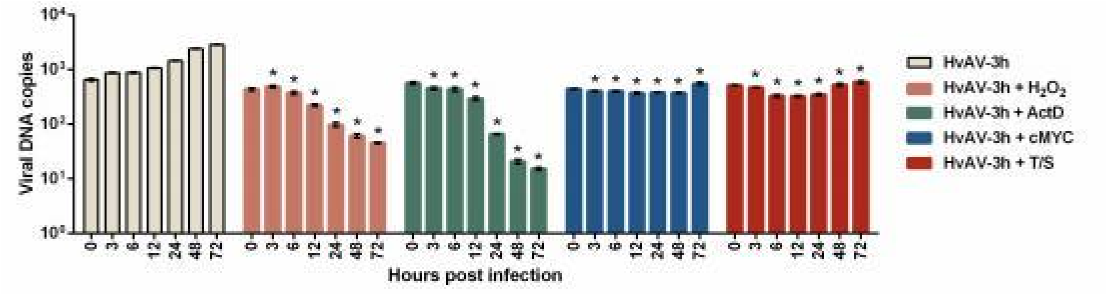

Supplement: FIG S1 [file mbio.03119-22-s0001.pdf]
