# Supplementary figures and images for: Apoptosis or Antiapoptosis? Interrupted Regulated Cell Death of Host Cells by Ascovirus Infection In Vitro
Source: mBio. 2023 Feb 6;14(1):e03119-22. doi: 10.1128/mbio.03119-22 (PMC9973268; doi:10.1128/mbio.03119-22)

# Supplementary Figures

**Figure S2**

The uncut immunoblotting images of Figure 7A.

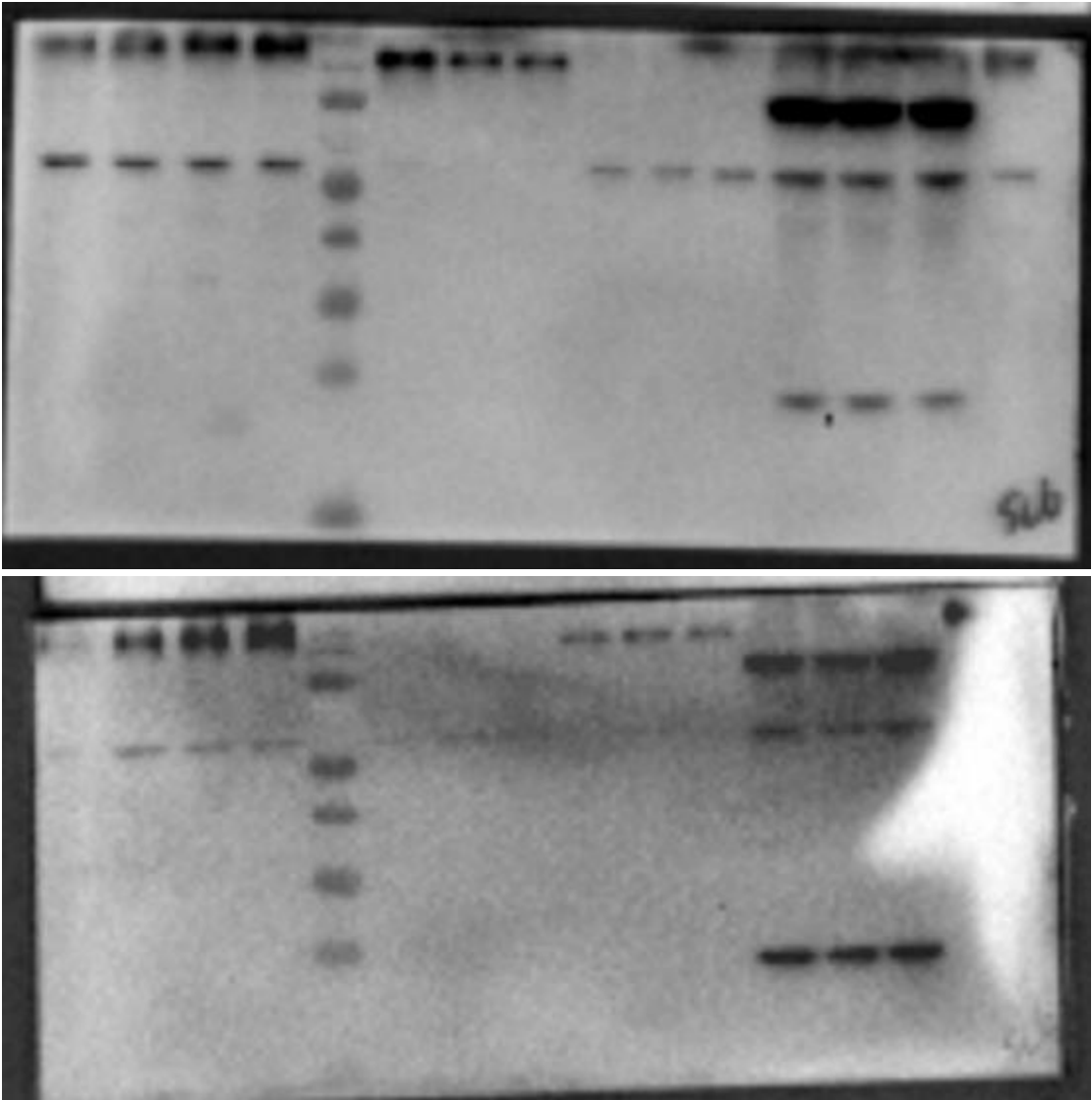

Supplement: FIG S2 [file mbio.03119-22-s0002.pdf]
